# Cavitary Pulmonary Nodules Associated With Leflunomide Therapy in Rheumatoid Arthritis: A Case Report and Literature Review

**DOI:** 10.7759/cureus.105449

**Published:** 2026-03-18

**Authors:** Demir Bedak, Rifat Sejdinovic, Besim Prnjavorac, Halida Hakic-Beslagic, Ajdin Ibrahimovic, Anel Mahmutovic, Omer Bedak

**Affiliations:** 1 Department of Pulmonology, General Hospital Tešanj, Tešanj, BIH; 2 Department of Pathology, General Hospital Tešanj, Tešanj, BIH; 3 Department of Internal Medicine, General Hospital Tešanj, Tešanj, BIH

**Keywords:** cavitary pulmonary nodules, drug-induced lung disease, extra-articular manifestations, leflunomide, rheumatoid arthritis

## Abstract

Rheumatoid arthritis (RA) may present with extra-articular pulmonary manifestations, including rheumatoid lung nodules. Leflunomide is an effective disease-modifying antirheumatic drug, but increasing case reports suggest an association with accelerated pulmonary nodulosis. We describe a 61-year-old woman with long-standing seropositive RA who developed left-sided lower chest pain, generalized weakness, fatigue, and loss of appetite after switching from methotrexate to leflunomide 20 mg/day. CT imaging demonstrated multiple bilateral cavitary nodules predominantly in the lower lobes. Extensive evaluation (bronchoscopy with microbiology, QuantiFERON-TB Gold, and malignancy assessment) was negative, and video-assisted thoracoscopic surgery lung biopsy showed necrotizing granulomatous inflammation consistent with rheumatoid nodules. The leflunomide dose was initially reduced and then discontinued, and methotrexate was reintroduced; at the four-month follow-up, the patient was clinically well without synovitis, and a CT eight months later showed near-complete regression of prior nodules, with residual left basal fibrotic changes containing calcifications and a remaining non-cavitary right lower-lobe nodule. Clinicians should consider leflunomide-associated pulmonary nodulosis in RA patients with new cavitary nodules after excluding infectious and malignant etiologies, and discontinuation of the drug may lead to radiologic improvement.

## Introduction

Rheumatoid arthritis (RA) is a common chronic systemic inflammatory disorder that mainly affects the joints. The ongoing systemic inflammation may also lead to several extra-articular manifestations, such as pulmonary involvement, rheumatoid vasculitis, and hematological abnormalities [1]. Peripheral rheumatoid nodules are a common and highly specific dermatologic feature of RA, while pulmonary nodules are a comparatively rare manifestation [2]. The reported prevalence of rheumatoid pulmonary nodules, also known as 'necrobiotic nodules,' varies from less than 0.4% in radiologic studies to as high as 32% in lung biopsy samples from patients with RA [3]. Pulmonary nodules are usually asymptomatic, but when they develop cavitation, they may cause complications such as pleural effusion or bronchopleural fistulas [4].

Leflunomide is a widely used disease-modifying antirheumatic drug (DMARD) that inhibits de novo pyrimidine synthesis by blocking the mitochondrial enzyme dihydroorotate dehydrogenase, thereby reducing lymphocyte proliferation. Additionally, it interferes with kinase activity, which is crucial for immune and inflammatory responses, and suppresses gene expression. Leflunomide also influences B-cell proliferation, neutrophil chemotaxis, and immunoglobulin production [5,6]. While leflunomide remains an effective and widely utilized therapy for RA, growing evidence suggests a possible association between its use and the development of pulmonary nodules.

In this case report, we present a clinically significant case of leflunomide-associated cavitary pulmonary nodules in a patient with seropositive RA and a near-complete CT resolution following drug discontinuation, supporting a likely association with leflunomide exposure.

## Case presentation

A 61-year-old woman with seropositive RA presented to the Department of Pulmonology at General Hospital Tešanj with complaints of left-sided lower chest pain, generalized weakness, fatigue, and loss of appetite. Her medical history was significant for arterial hypertension, osteoporosis, and chronic tobacco use for 43 years, with a cumulative exposure of 65 pack-years. She had been diagnosed with RA 14 years earlier. The disease was non-erosive but associated with deformities of the small joints. Her arthritis had been well controlled with methotrexate 15 mg weekly from 2011 until 2021, when the regimen was switched to leflunomide 20 mg daily due to nausea and abdominal pain. Her symptoms began approximately four years after starting leflunomide.

At the time of presentation, her regular medications included leflunomide 20 mg once daily, vitamin D 2000 IU daily, calcium 1000 mg daily, valsartan 80 mg daily, bisoprolol 2.5 mg daily, and intravenous ibandronic acid administered every three months. On examination, her blood pressure was 120/80 mmHg, heart rate 90 beats per minute, respiratory rate 15 breaths per minute, and temperature 36.6°C. She appeared well-nourished, without pallor or cyanosis. Cardiopulmonary examination was unremarkable. Physical examination revealed no peripheral rheumatoid nodules. 

**Figure 1:**
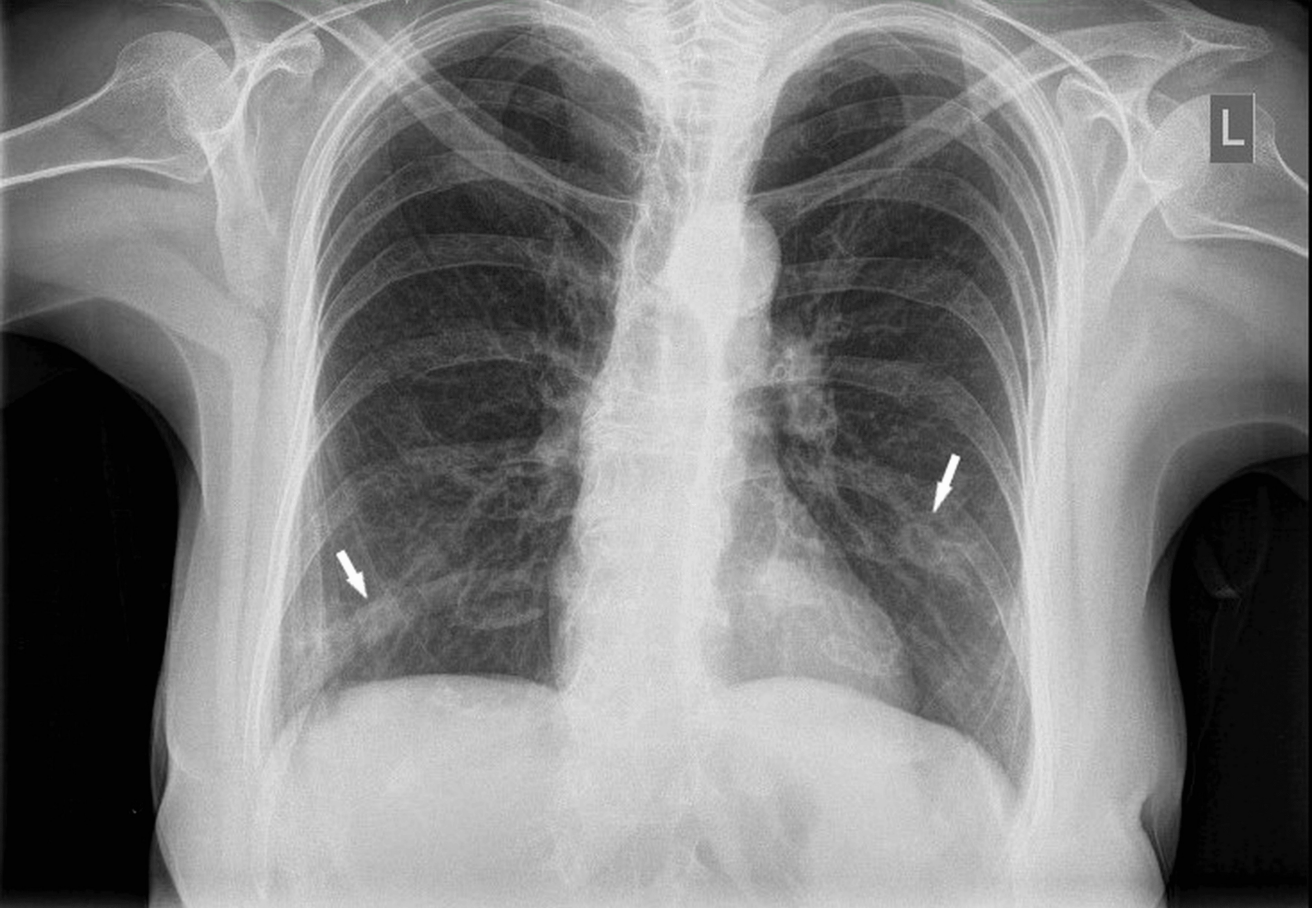
Posteroanterior chest radiograph showing bilateral cavitary pulmonary nodules (white arrows)

A posteroanterior chest radiograph showed bilateral cavitary pulmonary nodules (Figure 1).

Laboratory evaluation revealed an elevated ESR and D-dimer level, while all other parameters were within normal reference ranges. Tumor markers were within normal ranges (CA 19-9, CA 125, and CA 15-3), while CEA was slightly elevated (Table 1).

**Table 1:** Laboratory parameters of the patient after admission to the Pulmonology Department CRP: C-reactive protein; ESR: erythrocyte sedimentation rate; MCV: mean corpuscular volume; aPTT: activated partial thromboplastin time; PT: prothrombin time; INR: international normalized ratio; AST: aspartate aminotransferase; ALT: alanine aminotransferase; ALP: alkaline phosphatase

Parameter	Value	Reference range	Parameter	Value	Reference range
CRP	5.0 mg/L	< 5 mg/L	Urea	3 mmol/L	2.50 - 7.50 mmol/L
ESR	32 mm/h	4-15 mm/h	Creatinine	66 umol/L	53 - 101 umol/L
Glucose	5.9 mmol/L	4.1-6.1 mmol/L	Troponin I	2.4 ng/L	< 100 ng/L
Leukocytes	9.6 x 10^9/L	4.0-10.0 x 10^9/L	D-dimer	1388 ng/mL	< 500 ng/mL
Erythrocytes	4.02 x 10^12/L	3.50 - 5.50 x 10^12/L	AST	29 U/L	8 - 30 U/L
MCV	86.4 fL	80.00 – 100.00 fL	ALT	19 U/L	12 - 48 U/L
Hemoglobin	120 g/L	110 - 180 g/L	ALP	56 U/L	64 - 153 U/L
Platelets	290 x 10^9/L	150 - 500 x 10^9/L	CA 125	21 U/mL	< 35 U/mL
aPTT	35 s	29 - 42 s	CA 19-9	2 U/mL	< 37 U/mL
PT	17.1 s	12.0 – 18.0 s	CA 15-3	16 U/mL	< 30 U/mL
INR	1.16	0.96 – 1.18	CEA	18 ng/mL	<5 ng/mL

Two sputum samples were negative for acid-fast bacilli, mycology, and routine bacterial cultures. The QuantiFERON-TB Gold test was also negative. C and p-antineutrophil cytoplasmic antibodies (ANCAs) were also negative.

**Figure 2:**
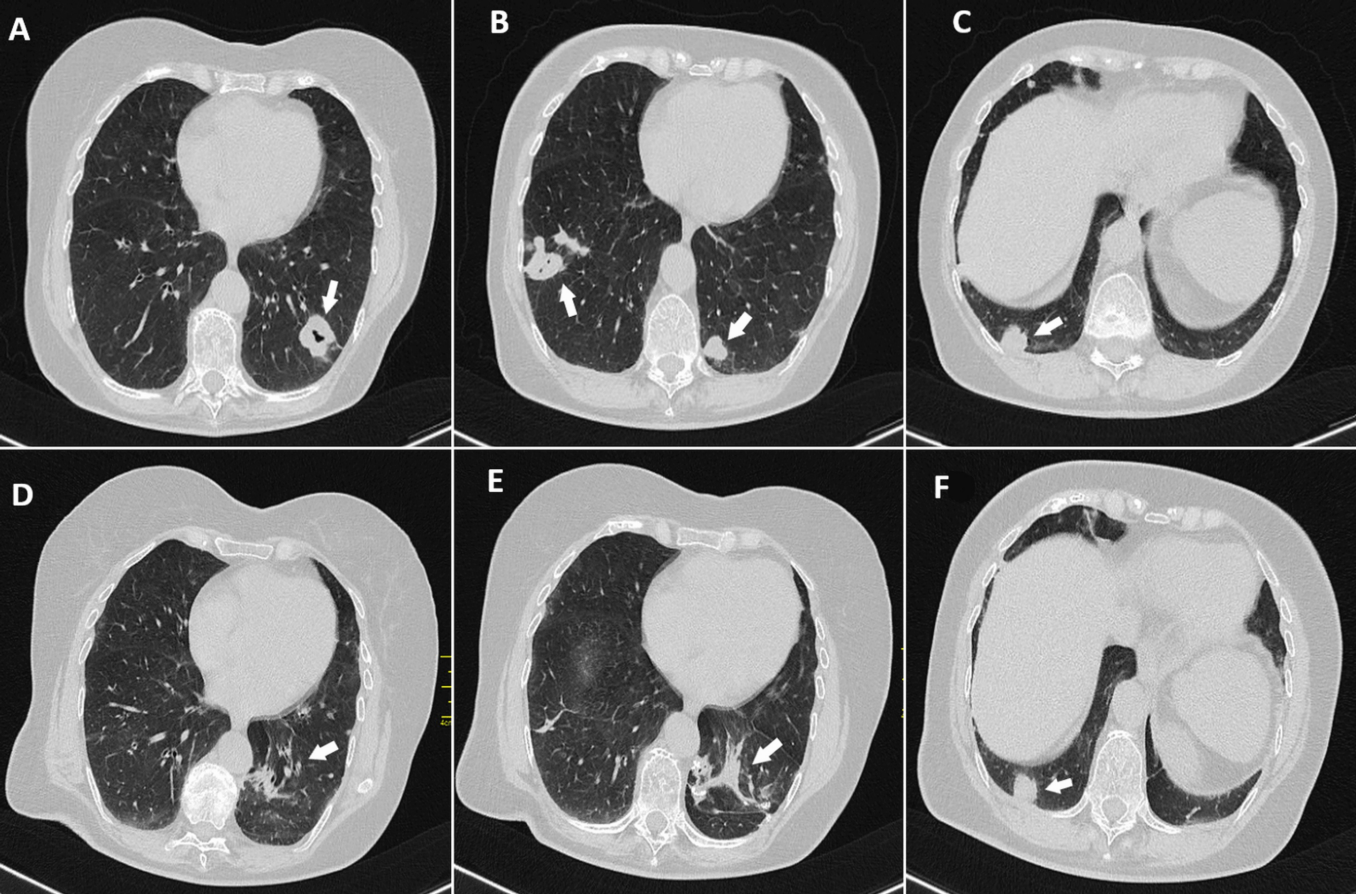
Contrast-enhanced chest CT scan (axial lung window) after hospital admission (A-C), and follow-up CT thorax performed eight months after the previous examination (D-F) (A) Cavitary nodule in the left lower lobe measured 36 × 26 mm. (B) Cavitary nodule in the right lower lobe measured 28 × 20 mm, as well as a solid nodule in the left lower lobe. (C) Solid nodule in the right lower lobe. (D-E) Left basal fibrotic changes containing calcifications. (F) A remaining solid nodule in the right lower lobe.

Further ultrasound examination of the neck, axillary regions, breasts, abdomen, and inguinal areas revealed no abnormalities. Contrast-enhanced chest CT imaging demonstrated multiple bilateral soft-tissue micronodules, nodules, and masses, predominantly distributed in the lower lobes, several of which exhibited central cavitation. The largest lesion measured 36 × 26 mm in the left lower lobe and 28 × 20 mm in the right lower lobe. Additional CT findings included signs of pulmonary emphysema, mild mediastinal and hilar lymphadenopathy, and hepatic steatosis without signs of pulmonary thromboembolism (Figures 2A-2C).

Bronchoscopy findings were unremarkable. Bronchoalveolar lavage (BAL) tested negative for acid-fast bacilli, while BAL cultures for mycobacteria and fungi showed no growth, and cytological examination revealed no evidence of malignant cells.

**Figure 3:**
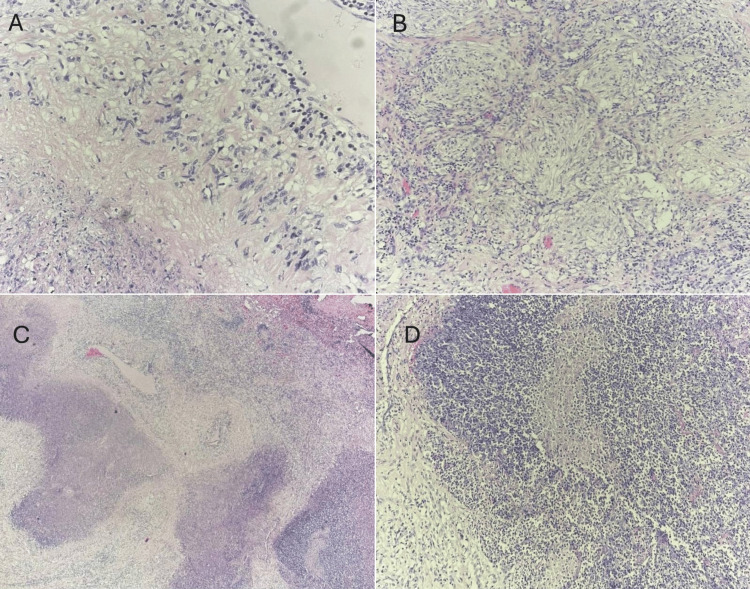
Lung biopsy histology (A) Rheumatoid nodule, showing the characteristic palisading granuloma with a core consisting of necrotic collagen and fibrin (stain: hematoxylin and eosin; original magnification x400). (B) Epithelioid granuloma and surrounding fibrosis (stain: hematoxylin and eosin; original magnification x200). (C) Rheumatoid nodules (stain: hematoxylin and eosin; original magnification x100). (D) Rheumatoid nodule with central fibrinoid necrosis (stain: hematoxylin and eosin; original magnification x400).

The patient underwent video-assisted thoracoscopic lung biopsy, where a pathohistological exam revealed necrotizing granulomatous inflammation, morphologically consistent with rheumatoid nodules (Figure 3). No fungi or acid-fast bacilli were identified. These findings were in line with the patient's long-standing RA and the recent transition in therapy from methotrexate to leflunomide.

Based on these results, which excluded both neoplastic and infectious causes, a rheumatologist was consulted and recommended tapering leflunomide to 20 mg every other day for one additional month, followed by complete discontinuation. The patient was restarted on methotrexate at a dose of 15 mg per week and folic acid, 5 mg (24 hours after the methotrexate).

At the four-month follow-up visit, the patient reported feeling well, with no clinical evidence of synovitis on joint examination.

A follow-up CT of the thorax performed eight months after the previous examination demonstrated left basal fibrotic changes containing calcifications, measuring approximately 65 × 33 mm, as well as a remaining non-cavitary nodule in the right lower lobe basally. Overall, there was near-complete regression of the previously described pulmonary nodules (Figures 2D-2F).

## Discussion

Pulmonary manifestations of RA encompass a broad spectrum of conditions, including interstitial lung disease, pleural effusion, bronchiectasis, bronchiolitis, and pulmonary nodules [7]. Among these, rheumatoid pulmonary nodules represent a relatively rare but well-recognized extra-articular manifestation, often associated with long-standing seropositive disease, male gender, and smoking [8]. The pathogenesis of rheumatoid nodules involves immune complex deposition, complement activation, and subsequent granulomatous inflammation leading to central necrosis and peripheral fibrosis [9]. Subcutaneous rheumatoid nodules are common in clinical practice, but pulmonary involvement occurs in less than 1% of patients on imaging studies, although histological evidence suggests a higher prevalence [8]. One of the proposed mechanisms linked to leflunomide-associated nodulosis is reduced monocyte activity, which consequently leads to impaired clearance of rheumatoid factor (RF) and, ultimately, to the accumulation of RF within the reticuloendothelial system, particularly in alveolar macrophages, where it may serve as a nidus promoting rheumatoid nodule formation [2]. Table 2 summarizes the published case reports of leflunomide-associated pulmonary nodules in patients with RA.

**Table 2:** Review of the 12 reports with 17 patients of leflunomide-associated pulmonary nodules from 2006 to 2024 M: male; F: female; NM: not mentioned; RN: rheumatoid nodule; GGOs: ground glass opacities; PNTX: pneumothorax; INF: infection

References	Case No.	Age	Sex	Smoking status	RA duration	Serological status	Leflunomide Dose	Leflunomide Duration	Radiological finding	Biopsy findings	Management	Outcome
Rozin et al. (2006) [10]	1	77	M	Smoker	18 years	Seropositive	20 mg/day	13 months	Several cherry-like nodes, partially with cavitation	RN	Leflunomide withdrawn	Stabilized
Rozin et al. (2006) [10]	2	66	M	Smoker	22 years	Seropositive	20 mg/day	7 months	Small lung nodes in the right lower lobe, partially with cavitation	RN	Leflunomide withdrawn	Stabilized
Horvath et al. (2008) [11]	-	34	F	Nonsmoker	9 years	Seropositive	20 mg/day	3 years	Multiplex GGOs bilateral	RN	Leflunomide withdrawn	Regression
Patel et al. (2009) [12]	-	62	F	Smoker	10 years	Seropositive	20 mg/day	10 years	Multiple cavitating lung nodules, bilateral	RN	Leflunomide withdrawn	Regression
Kim and Yoo (2011) [13]	-	46	F	Nonsmoker	4 years	Seropositive	20 mg/day	4 years	Right PNTX with multiple bilateral subpleural cavitary nodules	RN	Leflunomide withdrawn	Stabilized
Yoshikawa et al. (2015) [14]	-	60	F	Nonsmoker	10 years	Seropositive	20 mg/day	8 years	Multiple cavitary pulmonary nodules, predominantly in the left lung base	RN	Leflunomide withdrawn	Regression
Balkarli and Cobankara (2016) [4]	1	60	F	Smoker	5 years	Seropositive	20 mg/day	20 months	Multiple nodules in the lower basal segment of the right lung	RN	NM	NM
Balkarli and Cobankara (2016) [4]	2	42	F	Nonsmoker	20 years	Seropositive	20 mg/day	13 months	Bilateral multiple nodules at the posterior basal and laterobasal segments, cavitary	RN	NM	NM
Balkarli and Cobankara (2016) [4]	3	42	F	Nonsmoker	9 years	Seropositive	20 mg/day	15 months	Pleural effusion and 4 cavitary nodular lesions below the posterior peripheral segment	RN	NM	NM
Balkarli and Cobankara (2016) [4]	4	65	M	Nonsmoker	14 years	Seropositive	20 mg/day	10 months	Multiple cavitary nodules in the right lobe	RN	NM	NM
Kanıtez et al. (2018) [15]	1	55	F	Nonsmoker	20 years	Seropositive	20 mg/day	15 years	Bilateral multiple nodular lesions with extensive cavitary areas, especially in the lower zones	RN	Leflunomide withdrawn	Regression
Kanıtez et al. (2018) [15]	2	61	F	Nonsmoker	2 years	Seropositive	20 mg/day	10 months	Cavitary pulmonary nodules, PNTX	RN	Leflunomide withdrawn	Stabilized
Wickrematilake (2020) [16]	-	65	F	Nonsmoker	9 years	Seropositive	20 mg/day	NM	Multiple thick-walled cavitary lung nodules, bilateral	RN	Leflunomide withdrawn	Stabilized
Koslow et al. (2020) [17]	-	48	F	Nonsmoker	19 years	Seropositive	20 mg/day	3 years	Bilateral, asymmetrical, parenchymal, and subpleural lung nodules	Not performed	Leflunomide withdrawn	Regression
Bektyrganova et al. (2023) [18]	-	49	M	Nonsmoker	5 years	Seropositive	20 mg/day	3 years	Multiple diffuse nodular mass lesions of a round shape with sharp borders and homogeneous structure	RN	Leflunomide withdrawn	NM
Abiyarghamsari and Ahmadzadeh (2024) [2]	-	65	M	Smoker	20 years	Seropositive	20 mg/day	NM	Multiple cavitary pulmonary nodules, predominantly in the left lung base	INF	Leflunomide withdrawn	Regression
Coulibaly et al. (2024) [19]	-	58	F	Nonsmoker	21 years	Seropositive	20 mg/day	9 years	Bilateral excavated nodules	No malignancy	Leflunomide withdrawn	Regression

A total of 17 patients from 12 published case reports of leflunomide-associated pulmonary nodules or cavitary lung lesions (2006-2024) were identified. The mean age was 56 years, with 70.6% female patients. Smoking status was reported in most cases: 29.4% were smokers and 70.6% non-smokers, indicating that smoking was not a consistent contributor to nodulosis. The RA duration before nodule development ranged from 2 to over 30 years (median 12.8), and all cases were seropositive. Leflunomide was given at 20 mg/day in nearly all reports, with a median latency of 4.1 years before nodule appearance. Radiologically, cavitary nodules were most common (76.5%), often multiple, bilateral, and parenchymal or subpleural; non-cavitary lesions appeared in 23.5%. Occasional findings included ground-glass opacities, pleural effusion, or pneumothorax. Biopsy, when performed, showed rheumatoid nodules with necrobiotic granulomatous inflammation in 82.4% of cases; one report did not include a biopsy, and the remaining cases reported infection or exclusion of malignancy. Leflunomide withdrawal was the main intervention (70.6%), and among those who stopped the drug, 41.2% experienced partial or complete regression of nodules, while 29.4% showed stabilization. No progression was reported following withdrawal, although some cases lacked follow-up (Tables 2, 3).

**Table 3:** Summary of reported cases of leflunomide-associated pulmonary nodules in rheumatoid arthritis

Characteristic	Value
Number of cases	17
Age, years	56.2 ± 11.1
Sex, male	5/17 (29.4%)
Sex, female	12/17 (70.6%)
Smoking status: smoker	5/17 (29.4%)
Smoking status: nonsmoker	12/17 (70.6%)
Rheumatoid arthritis duration, years	Median 12.8
Serological status, seropositive	17/17 (100%)
Leflunomide dose	20 mg/day in 17/17 cases (100%)
Time on leflunomide, years	Median: 4.16
Radiology, cavitary lesions	13/17 (76.5%)
Radiology, non-cavitary lesions	4/17 (23.5%)
Biopsy finding: rheumatoid nodules (RN)	14/17 (82.4%)
Biopsy not performed	1/17 (5.9%)
Biopsy finding, inflammatory changes	1/17 (5.9%)
Biopsy finding, no malignancy	1/17 (5.9%)
Management, leflunomide withdrawn	12/17 (70.6%)
Management, not mentioned	5/17 (29.4%)
Outcome, regression	7/17 (41.2%)
Outcome, stabilized	5/17 (29.4%)
Outcome, not mentioned	5/17 (29.4%)

In RA, cavitary pulmonary nodules require careful exclusion of mycobacterial and fungal infection; septic pulmonary emboli; vasculitis; primary lung cancer or metastases; and inflammatory etiologies such as granulomatosis with polyangiitis and sarcoidosis, in addition to rheumatoid nodules. In our case, these conditions were excluded through radiologic assessment, bronchoscopy, microbiological testing, QuantiFERON analysis, ANCAs, tumor marker evaluation, and surgical biopsy, which served as the final confirmation of diagnosis. Tumor marker values CA 125, CA 19-9, and CA 15-3 were within the normal range, while CEA was elevated; however, CEA can be increased in smokers and in RA as well and therefore requires interpretation in a clinical context [20,21].

The most common management approach is discontinuation of leflunomide, which leads to regression of the pulmonary nodules in almost half of the cases (Tables 2, 3). Medications that have been linked to the formation of rheumatoid nodules include methotrexate, leflunomide, and anti-TNF-α agents [19]. In our case, the patient had previously received methotrexate and was later switched to leflunomide; the pulmonary nodules developed after initiation of leflunomide and showed near-complete resolution following drug discontinuation, supporting a likely association with leflunomide exposure.

To date, due to limited clinical evidence, there are no official guidelines for the treatment of rheumatoid nodules; however, cases with favorable outcomes have been reported. Biological agents such as tocilizumab and rituximab have been reported to facilitate regression of rheumatoid pulmonary nodules in some patients [16]. Braun and Wagener reported a retrospective series of 16 RA patients where rituximab was associated with regression of peripheral and pulmonary rheumatoid nodules. Most patients had long-standing, seropositive disease and extensive DMARD/biologic exposure. Clinically, nodules completely resolved in 6/16 patients, including the disappearance of pulmonary nodules in two cases, while 10/16 showed a ~50% reduction in nodule size [22]. Similar results were reported by Glace et al., who described outcomes with the anti-CD20 monoclonal antibody rituximab in 10 patients with pulmonary rheumatoid nodules, reporting an overall reduction in nodule size, with complete radiologic resolution in one patient [23]. Rozin et al. described administering cholestyramine (8 grams three times daily for a total of 11 days) to accelerate elimination, given its long half-life (approximately two weeks) and the time required to reach steady state (approximately 20 weeks) [10,24]. Therefore, cholestyramine washout may be considered in selected cases, especially in progressive disease, severe symptoms, or when rapid elimination is desired.

Although the reported outcomes are encouraging, the role of specific medications in the treatment strategy remains uncertain and requires further study.

## Conclusions

This case report highlights the diagnostic complexity of leflunomide-associated cavitary pulmonary nodules and contributes to the existing but limited literature. In patients with long-standing, seropositive RA, the development of multiple cavitary lung nodules requires precise evaluation for infection and malignancy; however, clinicians should also consider drug-associated pulmonary nodulosis, especially in those receiving leflunomide. Our case supports a clinically important association through histologic confirmation and a favorable regression of cavitary nodules after leflunomide discontinuation. Early recognition may prevent unnecessary prolonged antimicrobial treatment and support a management approach centered on excluding alternative etiologies, stopping leflunomide, and documenting response with serial imaging.
